# Mutations in the Phenicol Exporter Gene *fexA* Impact Resistance Levels in Three Bacterial Hosts According to Susceptibility Testing and Protein Modeling

**DOI:** 10.3389/fmicb.2021.794435

**Published:** 2022-01-07

**Authors:** Anja Müller, Keisuke Sakurai, Diana Seinige, Kunihiko Nishino, Corinna Kehrenberg

**Affiliations:** ^1^Institute for Veterinary Food Science, Justus Liebig University Giessen, Giessen, Germany; ^2^Institute for Protein Research, Osaka University, Osaka, Japan; ^3^Lower Saxony State Office for Consumer Protection and Food Safety, Wardenburg, Germany; ^4^SANKEN, Institute of Scientific and Industrial Research, Osaka University, Osaka, Japan

**Keywords:** antimicrobial resistance, exporter, transformation, susceptibility testing, *Staphylococcus aureus*

## Abstract

The prototype *fexA* gene confers combined resistance to chloramphenicol and florfenicol. However, *fexA* variants mediating resistance only to chloramphenicol have been identified, such as in the case of a *Staphylococcus aureus* isolate recovered from poultry meat illegally imported to Germany. The effects of the individual mutations detected in the *fexA* sequence of this isolate were investigated in this study. A total of 11 *fexA* variants, including prototype *fexA* and variants containing the different previously described mutations either alone or in different combinations, were generated by on-chip gene synthesis and site-directed mutagenesis. The constructs were inserted into a shuttle vector and transformed into three recipient strains (*Escherichia coli*, *Staphylococcus aureus*, and *Salmonella* Typhimurium). Subsequently, minimal inhibitory concentrations (MIC) of florfenicol and chloramphenicol were determined. In addition, protein modeling was used to predict the structural effects of the mutations. The lack of florfenicol-resistance mediating properties of the *fexA* variants could be attributed to the presence of a C110T and/or G98C mutation. Transformants carrying *fexA* variants containing either of these mutations, or both, showed a reduction of florfenicol MICs compared to those transformants carrying prototype *fexA* or any of the other variants. The significance of these mutations was supported by the generated protein models, indicating a substitution toward more voluminous amino-acids in the substrate-binding site of FexA. The remaining mutations, A391G and C961A, did not result in lower florfenicol-resistance compared to prototype *fexA*.

## Introduction

Phenicols are potent broad-spectrum antimicrobials that are effective against a range of pathogenic bacteria. However, the clinical use of chloramphenicol is severely limited owing to the possibility of severe side effects (Dinos et al., 2016; Li et al., 2020). The chloramphenicol derivative florfenicol shows reduced toxicity compared to chloramphenicol and remains active against bacteria that have acquired a *cat* gene, rendering them resistant to chloramphenicol (Dinos et al., 2016). To date, however, florfenicol is exclusively approved for use in veterinary medicine, where it is used to treat respiratory infections and other diseases in livestock and fish farming (Li et al., 2020). Acquired resistances to florfenicol have been identified in several bacterial species. The *fexA* gene encodes a protein with 14-transmembrane domains, functioning as an efflux pump of the major facilitator superfamily (Kehrenberg and Schwarz, 2004). This superfamily comprises a large number of different transport proteins which function as antiporters, symporters or uniporters utilizing the H^+^-gradient across the bacterial cell membrane (Kumar et al., 2020). While some members of this superfamily function as multidrug efflux pumps, *fexA* confers resistance exclusively to chloramphenicol and florfenicol. It is inducibly expressed and both florfenicol and chloramphenicol can effectively act as inducers (Kehrenberg and Schwarz, 2004). The *fexA* gene was first discovered in a *Mammaliicoccus* (formerly *Staphylococcus*) *lentus* isolate in 2003 and was shown to be part of a transposon, Tn*558*, located on a plasmid (Kehrenberg and Schwarz, 2004). It was subsequently detected in various staphylococcal species as well as in other Gram-positive bacteria such as enterococci, streptococci and a *Bacillus* sp. (Dai et al., 2010; Wang et al., 2013; He et al., 2016). However, it was shown to also be functional in *Escherichia coli* when introduced into this species (Kehrenberg and Schwarz, 2004). In staphylococci, it is often located together with *cfr*, which is of particular concern in clinical isolates due to the multidrug-resistance phenotype of such isolates (Kehrenberg and Schwarz, 2006; Li et al., 2018). More recently, a *fexA* variant was detected in a florfenicol-susceptible *S. aureus* isolate, recovered from poultry meat illegally imported into the EU. Sequence analyses of this *fexA* gene revealed four mutations resulting in amino acid substitutions in the deduced FexA protein (Müller et al., 2016). A similar variant of *fexA*, sharing three of the four mutations, was detected in a florfenicol-susceptible *S*. *pseudintermedius* isolate (Gómez-Sanz et al., 2013). The reversion of two mutations in this variant was reported to restore the florfenicol-resistance phenotype in a previously susceptible *E. coli* recipient (Gómez-Sanz et al., 2013). However, the potential influence on florfenicol resistance levels of the remaining amino acid substitutions present in the *fexA*-variants has not been examined. In addition, previous research was limited to *E. coli* and the effects in staphylococcal isolates have not been established yet. The current study aims to evaluate the role of the individual mutations and their respective combinations on the resistance-mediating properties of *fexA*, in Gram-negative as well as Gram-positive recipient strains.

## Materials and Methods

### Construction of *fexA* Variants and Recombinant Vectors

This study was based on a variant of the *fexA* gene we have previously identified in a *S. aureus* isolate recovered from poultry meat illegally imported to Germany from Egypt. This variant featured a total of four mutations (G98C, C110T, A391G, C961A; GenBank: KX230476), compared to prototype *fexA* (Müller et al., 2016). In order to elucidate the effect of these mutations, *fexA* variants were created featuring each of these mutations either individually or in different combinations. In addition, a similar *fexA* variant previously detected in a florfenicol-susceptible isolate of *S*. *pseudintermedius* was included in the study (Gómez-Sanz et al., 2013). This variant shared three mutations with the *fexA* gene we detected in *S. aureus* but featured a C199A mutation instead of C961A (GenBank: HF679552). Lastly, we also included the prototype *fexA* gene for comparison. This resulted in a total of 11 *fexA* variants that were generated for further investigations in the course of this study (Table 1).

**TABLE 1:** MIC values observed in transformants of *S. aureus* RN4220, *E. coli* TOP10 and *Salmonella* Typhimurium ATCC14028s Δ*acrB* carrying the different *fexA* variants.

*fexA*-variant	MICs (μg/ml) in recipient strain					
	*S. aureus* RN4220		*E. coli* TOP10		*S.* Typhimurium ATCC14028s Δ*acrB*	
	FFN	CHL	FFN	CHL	FFN	CHL
None (empty recipient)	4	8	4	4	1	1
*fexA*	16	32	16	32	4	8
*fexA*_G98C	8	32	8	32	2	8
*fexA*_C110T	4	16	4	16	2	4
*fexA*_A391G	16	32	16	32	4	8
*fexA*_C961A	16	32	16	32	4	8
*fexA*_G98C + C110T	4	16	4	16	2	4
*fexA*_G98C + A391G	8	32	8	32	2	8
*fexA*_C110T + A391G	4	32	4	32	2	8
*fexA*_G98C + C110T + A391G	4	32	4	32	2	8
*fexA*_G98C + C110T + A391G + C199A	4	32	4	32	2	8
*fexA*_G98C + C110T + A391G + C961A	4	32	4	32	2	8

*FFN, florfenicol; CHL, chloramphenicol.*

**FIGURE 1:**
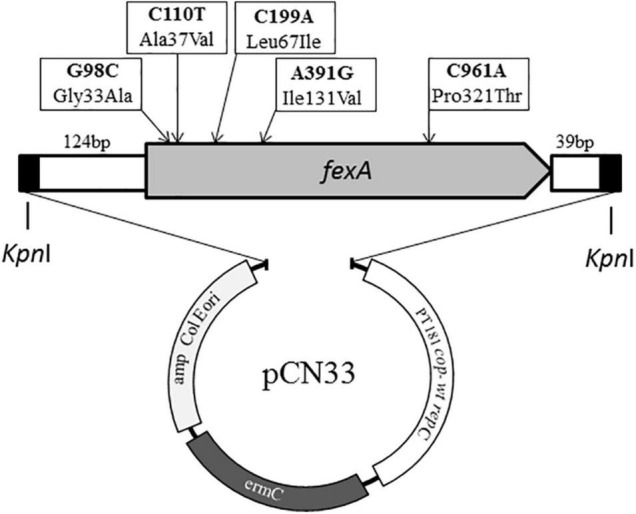
Schematic overview of the composition of synthesized *fexA* constructs and the vector pCN33 (Charpentier et al., 2004). The location of all mutations occurring among the different synthesized *fexA* variants are indicted with arrows.

First, the prototype *fexA* gene, known to mediate resistance to chloramphenicol and florfenicol (GenBank: AJ549214), was synthesized by on-chip gene synthesis and then 10 mutational variants of the gene were generated using site-directed mutagenesis (General Biosystems, Inc., Morrisville, NC, United States) (Quan et al., 2011). The final 1,603 bp inserts further included 124 bp upstream of *fexA*, containing its regulatory region, and 39 bp downstream of *fexA*, according to the published sequences, and it was flanked by *Kpn*I restriction sites on both ends (Figure 1). The gene constructs were inserted into the multiple cloning site of *E. coli* – staphylococcal shuttle vector pCN33, containing an erythromycin and an ampicillin resistance determinant for the selection of Gram-positive and Gram-negative transformants, respectively (General Biosystems, Inc., Morrisville, NC, United States) (Charpentier et al., 2004). The sequence of all inserts was confirmed by sequence analyses.

### Transformation Experiments

Three different recipient strains were used in this study. The recombinant vectors were transferred into *S. aureus* RN4220 by protoplast transformation (Kehrenberg and Schwarz, 2006). *S. aureus* transformants were selected using regeneration plates containing 20 μg/ml erythromycin, according to vector specifications. The recombinant vectors were further transferred into chemically competent *E. coli* TOP10 (Invitrogen™, Thermo Fisher Scientific, Waltham, United States) and by electroporation (Gene Pulser Xcell Electroporation System, Bio-Rad, Feldkirchen, Germany) into a *Salmonella* Typhimurium ATCC14028s mutant with a disrupted *acrB* gene, resulting in a multidrug efflux deficiency (Kehrenberg and Schwarz, 2001; Horiyama et al., 2010). Selection of Gram-negative transformants was performed using Luria Bertani (LB) agar plates containing 50 μg/ml ampicillin.

The presence of *fexA* in the respective transformants was subsequently confirmed by PCR with previously described primers (Kehrenberg and Schwarz, 2005).

### Antimicrobial Susceptibility Testing

Transformants were subjected to broth macrodilution susceptibility testing to determine MIC values for florfenicol and chloramphenicol. Testing was performed following Clinical and Laboratory Standards Institute (CLSI) standards for non-fastidious bacteria that grow aerobically [Clinical and Laboratory Standards Institute (CLSI), 2018]. Two-fold serial dilutions containing chloramphenicol (Carl Roth, Karlsruhe, Germany; purity ≥98.5%) or florfenicol (FLUKA brand, Sigma-Aldrich, Darmstadt, Germany; purity >99%) were prepared using cation-adjusted Mueller Hinton Broth (Becton Dickinson, Heidelberg, Germany). After tubes were prepared, they were inoculated immediately and incubated for 18–20 h at 35 ± 2°C before MIC values were determined.

Susceptibility testing was performed at least twice per transformant. *E. coli* ATCC25922, grown overnight on LB agar plates, was used as quality control strain and it was ensured that its MIC values were within the reference range of 2–8 μg/μl for both antimicrobials when evaluating test results (Clinical and Laboratory Standards Institute (CLSI), 2020, 2021).

### Modeling

The predicted structure model of FexA and variant FexA were calculated with the prototype and variant FexA amino acid sequence using AlphaFold v2.0 (Jumper et al., 2021) which was downloaded from GitHub^1^ on July 18, 2021. Variant FexA amino acid sequence contained Gly33Ala, Ala37Val, Leu67Ile, Ile131Val, and Pro321Thr mutations, which were all mutations in this study. Calculation options were set to “2020-07-14” for “max_template_date” and “full_dbs” for “preset.” 5 models of FexA and variant FexA were calculated and the highest pLDDT (predicted Local Distance Difference Test) score is 89.757 and 89.371, respectively. All models covered the 1–475 amino acid sequences and had 14-transmembrane α-helices, as previously noted (Kehrenberg and Schwarz, 2004). No significant difference was found among all results except for residues 1–19.

## Results and Discussion

### Effect of Mutations on Minimal Inhibitory Concentrations

In order to investigate the impact of mutations in the *fexA* florfenicol and chloramphenicol resistance gene observed in staphylococcal field isolates, a total of 11 variants were constructed and introduced into Gram-positive and Gram-negative recipient strains. An overview of the MIC values for chloramphenicol and florfenicol of the different *fexA* variants in the three different recipients is given in Table 1. As expected, all variants raised the MIC values of chloramphenicol in all three recipients (Table 1). For *E. coli* and *S. aureus* transformants, this resulted in either a resistant (MIC ≥ 32 μg/ml) or intermediate (MIC = 16 μg/ml) phenotype (Clinical and Laboratory Standards Institute (CLSI), 2021). Due to its impaired multidrug efflux, resulting from the disruption of the *acrB* gene (Horiyama et al., 2010), the MIC values for *S*. Typhimurium Δ*acrB* were overall lower than those observed for the other two recipients by 1–3 twofold dilution steps and remained within the susceptible range for chloramphenicol (MIC ≤ 8 μg/ml). Interestingly, transformants carrying the C110T mutation either alone or in combination with G98C only, showed a lower chloramphenicol MIC value compared to the remaining transformants. However, none of the variants featuring the A391G mutation in addition to C110T showed this reduction of chloramphenicol MICs. These results suggest a modest influence of the C110T mutation on the MIC value of chloramphenicol, which can be counterbalanced by an A391G substitution.

All transformants carrying the prototype *fexA* gene further showed a four-fold increase of their MIC value for florfenicol. The same MIC values were observed for transformants carrying either the A391G or the C961A mutations separately, demonstrating that the resulting Ile131Val and Pro321Thr amino acid exchanges do not play a role in the loss of florfenicol resistance. Lower florfenicol MIC values were observed exclusively in variants featuring a C110T and/or a G98C mutation. In *S. aureus* RN4220 and *E. coli* TOP10, the *fexA* variants featuring C110T did not mediate any elevation in florfenicol MICs, compared to the empty recipient strains. However, in *S*. Typhimurium ATCC14028s Δ*acrB*, a modest increase of the florfenicol MIC from 1 μg/ml to 2 μg/ml was observed. This indicates that the altered FexA proteins retain a low-level activity against florfenicol. The fact that the effect is only apparent in the ATCC14028s Δ*acrB* recipient is likely due to the overall higher base MIC values of the *S. aureus* and *E. coli* recipients (4 μg/ml), where the remaining activity of the FexA variants presumably does not suffice to result in a visible effect on florfenicol MICs.

The G98C mutation had a more moderate effect on florfenicol resistance. *E*. *coli* and *S*. *aureus* transformants carrying this mutation (but not C110T) in the *fexA* gene showed an increase of florfenicol MIC values of one twofold dilution step, compared to the empty recipient. This was the case for *fexA*^G98C^ and *fexA*^G98C+A391G^. All other variants containing G98G also featured the C110T mutation and thus mediated base level florfenicol MICs in *E. coli* and *S. aureus*.

### Modeling of FexA

**FIGURE 2:**
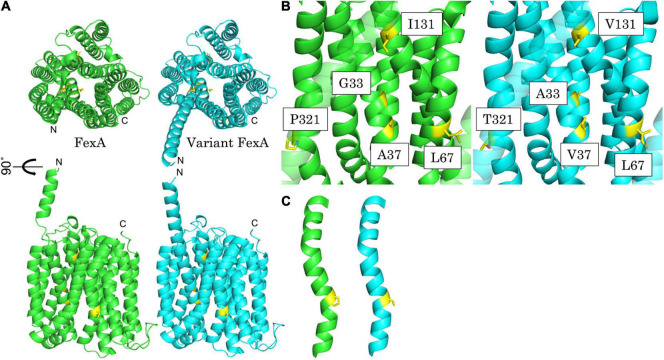
Best predicted model of FexA. **(A)** Calculated model of prototype FexA (green) and variant FexA (cyan) sequence from *S. aureus* W333 (GenBank KX230476). Mutated residues are painted yellow. Up, Upper view; Down, side view; Drawing range, Met1 – Gly475. **(B)** Detailed view of the mutated residues of the variant FexA sequence from *S. aureus* W333 and side by side comparison with prototype FexA. Left/green, prototype FexA; right/cyan, variant FexA. **(C)** Gradually curved α-helix included Pro321/Thr321. Mutated residues are painted yellow. Left/green, prototype FexA; right/cyan, variant FexA.

In order to explore the structural effects of the mutations underlying the observed differences in conferred phenotypes, protein modeling of FexA was performed (Figure 2). As the exact structure of FexA was unknown, we have calculated two predicted models using AlphaFold v2.0. Both models were transporters with a substrate-binding site at the center of the protein. The structure of the homology model suggested that mutations of G98C (Gly33Ala), C110T (Ala37Val), and A391G (Ile131Val) affect substrate binding, because they are located at the substrate-binding site. This prediction agreed well with the MIC values, except for A391G. Gly33Ala and Ala37Val were mutations toward more voluminous amino acids, which means the volume of the substrate-binding pocket is reduced. This would be expected to have a major impact on efflux activity. The determination of MIC values showed that both mutations decreased the affinity between FexA and florfenicol, however, the G98C (Gly33Ala) mutation did not result in a change in chloramphenicol MICs. This suggested that chloramphenicol did not interact with Gly33. The portion corresponding to the nitro group of chloramphenicol was replaced by a more voluminous methylsulfonyl group in florfenicol, and the side chain methyl group of Gly33Ala might collide physically. While the A391G (Ile131Val) mutation was located in the substrate-binding site, it did not lower the efflux activity of florfenicol and chloramphenicol. This was probably due to the fact that it was located far away from the binding position of both substrates. In addition, since isoleucine and valine have the same hydrophobic residue, it was presumed that the effect on the function of FexA was only small. The observed effect of A391G seemingly counteracting the slight reduction of chloramphenicol MICs by C110T could not be elucidated by the model. On the other hand, C199A (Leu67Ile) and C961A (Pro321Thr) were not located at the substrate binding site. Therefore, they are unlikely to have an effect on the efflux activity, which is in agreement with the observed MICs. The mutation from leucine to isoleucine by C199A likely had no effect because the volume and hydrophobicity of the amino acid did not change. In the predicted models, Pro321 was incorporated into a gently curved α-helix (Figure 2C). Since proline acted to bend the α-helix, Pro321Thr mutation was thought to form a linear α-helix and produce three-dimensional structural changes, which was suspected to have a very large influence on its function. However, calculated model of mutated FexA maintained a similar curved α-helix and three-dimensional structure. This is strongly correlated with no MIC difference in FexA Pro321 vs. Thr321.

### Comparison of *fexA* Variants From Different Hosts

Of the *fexA* variants artificially created in this study, some have already been demonstrated to occur naturally in different bacterial species, according to sequences deposited in the GenBank database^2^. This includes the *fexA*^G98C+C110T+A391G+C961A^ variant we have originally detected in *S. aureus*, which constituted the basis for the current study (Müller et al., 2016). According to a recent publication, this *fexA* variant was also detected in MRSA in Kuwait hospitals where the spread of closely related isolates carrying variant *fexA* was associated with an increased prevalence of chloramphenicol-resistance (Udo et al., 2021). A similar variant, *fexA*^G98C+C110T+A391^, was previously detected in a *Bacillus* sp. (GenBank: CP025122), however, no further data regarding the antimicrobial resistance phenotype of this isolate were published. The *fexA*^A391G^ variant was detected in a *S. cohnii* isolate (JF834909) as well as in a *S*. *epidermidis* isolate (KM521837). These isolates additionally carried the multidrug resistance gene *cfr*, which also confers resistance to phenicols (Wang et al., 2012; Bender et al., 2015). Consequently, the observed MIC values provided no precise information about the activity of these *fexA* variants.

In one previous study reporting a *fexA* variant detected in a florfenicol-susceptible *S. pseudintermedius* isolate, the gene was further analyzed (Gómez-Sanz et al., 2013). In that study, the reversion of the C110T and the G98C mutations of the *fexA* gene by site-directed mutagenesis was reported to restore the florfenicol-resistance phenotype in *E. coli* HB101 transformants (Gómez-Sanz et al., 2013). Transformants carrying the reverted positions separately, thus retaining either C110T or G98C, showed the same florfenicol MIC values as the empty recipient (Gómez-Sanz et al., 2013). According to these observations, both mutations resulted in a fully florfenicol-susceptible phenotype on their own. However, the effects of the mutations were only studied in an *E. coli* recipient and not in the genus *Staphylococcus*, in which the gene was detected. In contrast to these previous observations, the G98C mutation by itself did not result in base-level florfenicol MICs in any of the recipient strains used in the current study. In addition, we observed slight differences in the chloramphenicol MICs of transformant carrying *fexA* variants featuring the C110T mutation. However, we only observed this effect in variants lacking the A391G mutation, which was not reverted in the previous study (Gómez-Sanz et al., 2013). Consequently, the effect would not be expected to be seen in the *E. coli* HB101 transformants. Overall it should be noted that different recipient strains (including a highly susceptible strain and a Gram-positive species) were used in the current study, which may explain differences in observed MIC values compared to *E. coli* HB101.

In summary, this study demonstrated that the presence of C110T and G98C mutations in the *fexA* gene lower its resistance-mediating properties. The altered resistance-phenotype was substantiated with protein modeling, predicting a major impact of these mutations on efflux activity due to amino acid substitutions at the substrate-binding site.

## Data Availability Statement

The original contributions presented in the study are included in the article/supplementary material, further inquiries can be directed to the corresponding author/s.

## Author Contributions

CK conceived the initial project idea and coordinated the project. AM and CK designed *fexA* constructs. AM and DS performed transformation experiments and antimicrobial susceptibility testing. AM, DS, and CK analyzed the resulting data. KS and KN created and analyzed protein models of *fexA*. AM wrote the initial draft of the manuscript, with contributions by KS and KN. All authors shared in the revision and editing of the drafted manuscript, and approved the final version.

## Conflict of Interest

The authors declare that the research was conducted in the absence of any commercial or financial relationships that could be construed as a potential conflict of interest.

## Publisher’s Note

All claims expressed in this article are solely those of the authors and do not necessarily represent those of their affiliated organizations, or those of the publisher, the editors and the reviewers. Any product that may be evaluated in this article, or claim that may be made by its manufacturer, is not guaranteed or endorsed by the publisher.

## Footnotes

1
https://github.com/deepmind/alphafold

2
https://www.ncbi.nlm.nih.gov/genbank/
